# Influence of the magnetic nanoparticle coating on the magnetic relaxation time

**DOI:** 10.3762/bjnano.11.105

**Published:** 2020-08-12

**Authors:** Mihaela Osaci, Matteo Cacciola

**Affiliations:** 1 “Politehnica” University of Timisoara, Department of Electrical Engineering and Industrial Informatics, 2 Victoriei Square, 300006 Timisoara, Timis County, Romania; 2 Cooperativa TEC, Via Nazionale, n. 439, 89134 Pellaro di Reggio Calabria, Italy

**Keywords:** colloidal system, effective Verlet-type algorithm, magnetic relaxation time, nanoparticle coating, numerical simulation, stochastic Langevin dynamics method, superparamagnetic nanoparticles

## Abstract

Colloidal systems consisting of monodomain superparamagnetic nanoparticles have been used in biomedical applications, such as the hyperthermia treatment for cancer. In this type of colloid, called a nanofluid, the nanoparticles tend to agglomeration. It has been shown experimentally that the nanoparticle coating plays an important role in the nanoparticle dispersion stability and biocompatibility. However, theoretical studies in this field are lacking. In addition, the ways in which the nanoparticle coating influences the magnetic properties of the nanoparticles are not yet understood. In order to fill in this gap, this study presents a numerical simulation model that elucidates how the nanoparticle coating affects the nanoparticle agglomeration tendency as well as the effective magnetic relaxation time of the system. To simulate the self-organization of the colloidal nanoparticles, a stochastic Langevin dynamics method was applied based on the effective Verlet-type algorithm. The Néel magnetic relaxation time was obtained via the Coffey method in an oblique magnetic field, adapted to the local magnetic field on a nanoparticle.

## Introduction

One of the most important biomedical applications of colloidal magnetic nanoparticle systems is magnetic hyperthermia applied as an alternative for cancer treatment. Upon reaching the tumour, the magnetic nanoparticles are locally subjected to an alternating magnetic field, generating heat that kills the cancer cells [1]. The heat is generated due to two phenomena: Néel relaxation (an internal phenomenon driven by the rotation of the particle magnetic moment inside the particle) and Brown relaxation (an external phenomenon driven by the rotation of the nanoparticle along the magnetic moment). Both internal and external sources of friction lead to a delay in the orientation of the particle magnetic moment in the direction of the applied magnetic field, thus generating heat. This heat increases the tumour cell temperature which leads to cell death [1–4].

Iron-oxide magnetic nanoparticles, in particular magnetite (Fe_3_O_4_) and maghemite (γ-Fe_2_O_3_), have been intensely studied in the context of magnetic hyperthermia applications. These nanoparticles can be synthesized in small dimensions, which ensures low toxicity and the possibility for easy surface functionalization.

A common method for synthesising iron-oxide nanoparticles includes chemical co-precipitation, which involves the simultaneous precipitation of magnetic nanoparticles and a solid matrix through a sol–gel process, yielding metal-oxide nanoparticles dispersed in a mesoporous matrix. [5]. Other methods used for synthesising these nanoparticles include modifications of the sol–gel method. These methods involve supercritical conditions, such as ethyl alcohol and alkaline co-precipitation, and an additional step in which the hydrothermal method or thermal decomposition technique are used. The method used to obtain nanoparticles by thermal decomposition of an iron precursor in the presence of NaBH_4_ in a polyol was found to be suitable for size control in both chemical approaches [1–4,6].

Since the methods used to synthesise nanoparticles can affect their size, chemical composition and crystalline structure, special attention has been given to improving nanoparticle production quality. For example, the Plackett–Burman technique is a filtration method used for investigating the initial steps that influence the characteristics of the final material [7].

Uncoated superparamagnetic nanoparticles are difficult to synthesise since they are not stable in colloidal suspensions. Therefore, it is challenging to use these nanoparticles in magnetic hyperthermia therapy [8]. By exposing these nanoparticles to the acidic environment of living organisms, certain structural degradation processes occur due to the corrosion of nanoparticle surfaces. This biodegradation in acidic media leads to significant changes in the nanoparticle magnetic properties over time [9]. Since the nanoparticle surfaces are in direct contact with blood and other tissues, a biocompatible and nontoxic coating needs to be placed around the nanoparticles to prevent biodegradation processes. The coating thickness can significantly affect the magnetic properties and the hyperthermia of the nanoparticles. The coating is performed to reduce the sensitivity of nanoparticles to air, humidity and acidity. In addition, it allows for the functionalization and absorption of proteins and creation of hydrophilic molecules at the surface of the nanoparticles to prevent agglomeration, reducing capillary obstruction risk. Coating can also improve nanoparticle circulation in the blood and proper transport to the targeted areas, while preserving their physical–chemical properties. Additionally, coating prevents nanoparticle opsonisation by the reticuloendothelial system, which is pivotal for determining how fast nanoparticles can flow on the bloodstream before reaching their target.

The materials used as coating agents for magnetic nanoparticles can be organic or inorganic. The inorganic coating enables the surface of the nanoparticles to bind to their biological ligands, while maintaining the nanoparticle stability. On the other hand, organic coating (particularly polymers) has a number of advantages over inorganic coating, such as better particle dispersion, good colloidal stability, biocompatibility, good nanoparticle circulation in the blood, reduced toxicity and low risk of blood capillary obstruction.

In the last years, a new class of stable and biocompatible nanofluids have been developed by using a combination of electrostatic and steric stabilisation methods [10]. Despite these stabilization methods, a number of recent studies have experimentally shown a tendency for nanoparticle agglomeration, even in the absence of an external magnetic field [11–12]. This can be a potential problem when ferrofluids are used in medical applications, since nanoparticle agglomeration and sedimentation can create thrombi inside the blood vessels [13].

Controlling nanoparticle agglomeration is essential to improve the applicability of the magnetic nanoparticles. In this regard, the optimized microemulsion method can be used to obtain a homogenous silica coating on Fe_3−_*_x_*O_4_ nanoparticles [14]. This method controls the thickness of the coating layer, enabling a higher average separation among particles when compared to the oleic acid coating method used on pristine nanoparticles [14].

Homogeneous, polymer-coated spherical magnetite nanoparticles with superparamagnetic properties have been successfully synthesised. The polymer coating provides extra stability to the magnetic nanoparticles in aqueous media [15]. To increase biocompatibility or to enable specific hydrophilic properties, nanoparticles were coated with poly(ethylene glycol) (PEG) [16].

Experimental data concerning how different coatings influence nanoparticle magnetic properties are quite controversial. A few studies indicate that a thin polymer coating layer enhances the hyperthermia efficiency [17], while others do not suggest a correlation between the coating layer thickness and the magnetic hyperthermia properties (i.e., the absorption rate) [18].

These issues demonstrate the importance of investigating the ways in which the coating influences magnetic nanoparticle properties [8]. In order to solve these issues, the current study aims to use simulation models to study the influence of nanoparticle coating on nanoparticle agglomeration tendency and on the Néel magnetic relaxation time, as well as on the effective magnetic relaxation time.

## Results and Discussion

### Simulation methods used in the study

The agglomeration of magnetic nanoparticles evolves depending on the initial configuration of the system and on the specific parameters related to the nanoparticle coating. For each agglomeration state, the relaxation time is calculated with respect to the corresponding magnetic configuration of the system.

For the numerical simulation, two widely known models have been used [19–21]. We started with a system of single-domain magnetic nanoparticles, consisting of spherical iron-oxide nanoparticles with uniaxial magnetic anisotropy, which have a lognormal distribution of the grain size. Each nanoparticle is composed of a magnetic core and a nonmagnetic surface layer of stabilizing surfactant. The system temperature is considered to be constant.

To simulate the self-organization of the colloidal magnetic nanoparticles we used the Langevin dynamics stochastic method, based on an effective Verlet-type algorithm [19].

The Néel magnetic relaxation time 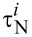 is obtained through the Coffey method in an oblique magnetic field, adapted to the local magnetic field of a nanoparticle [22–23].

For each nanoparticle, the effective magnetic relaxation time can be described as follows [24]:

[1]
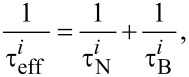


where 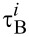 is the Brownian relaxation time. The Brownian process represents the nanoparticle rotation in the fluid environment. For spherical particles, the Brownian relaxation time is usually described as [24]:

[2]
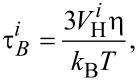


where 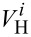 is the hydrodynamic volume of the *i*-th nanoparticle, η is the coefficient of dynamic viscosity, *k*_B_ is the Boltzmann constant, and *T* is the temperature.

After obtaining the effective magnetic relaxation time value of each nanoparticle, we can calculate the average effective magnetic relaxation time. The effective magnetic relaxation time is influenced by the magnetic nanoparticle coating. This influence is either due to the Brownian relaxation time (via the hydrodynamic volume, Equation 2), or due to the Néel relaxation time, via the nanoparticle configuration in the agglomerates, playing an important role in the calculation of the dipolar magnetic field acting on each particle [25].

The internal dipolar magnetic field is given as

[3]
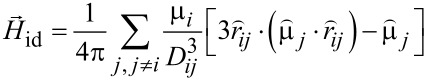


where *D_ij_* is the distance between the centres of those two nanoparticles, 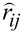 is the versor of the direction connecting the *i*-th and *j*-th nanoparticles, μ*_j_* is the magnetic moment of the *j*-th nanoparticle (
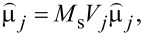
 where *V_j_* is the magnetic core volume of the *j*-th nanoparticle, *M*_s_ is the spontaneous magnetisation and 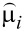 and 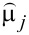 are the unit vectors of the magnetic moments of the *i*-th and *j*-th nanoparticles, respectively).

The local magnetic field acting on a nanoparticle is the vectorial sum of the applied external magnetic field (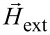) and the internal dipolar magnetic field (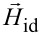) determined by the magnetic dipolar interactions among the nanoparticles [21],

[4]
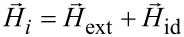


### Method for simulating the self-organization of colloidal magnetic nanoparticles

This method starts by obtaining the numerical solutions of the Langevin equations for the translational and rotational motions of a nanoparticle *i* in the fluid environment [19] given as

[5]
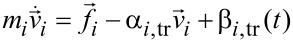


[6]
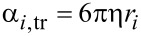


[7]
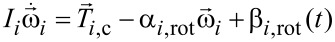


[8]
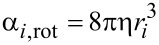


where *m_i_* is the mass of the *i*-th nanoparticle, 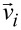 is the linear speed of the *i-*th nanoparticle, 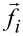 is the resultant of the conservative forces acting on the *i-*th nanoparticle, α*_i_*_,tr_ and α*_i_*_,rot_ are the translational and rotational friction coefficients, respectively, η is the dynamic viscosity coefficient, *r*_i_ is the radius of the *i-*th nanoparticle, β*_i_*_,tr_(*t*) and β*_i_*_,rot_(*t*) are the random Brownian force and torque, respectively, *I_i_* is the moment of inertia of the *i-*th nanoparticle, 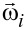 is the angular speed of the *i-*th nanoparticle, 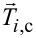 is the resultant of the conservative torques acting on the *i-*th nanoparticle.

The forces acting on the nanoparticles of the system are the van der Waals forces, electrostatic repulsive forces, magnetic dipolar forces, steric repulsion forces and the random Brownian force [19,26–29]. The stabilisation of magnetic particles can be achieved by the equilibrium between the electrostatic and steric repulsive forces [19,26,28].

The influence of nanoparticle coating on the nanoparticle interaction forces depends on the hydrodynamic dimension of the nanoparticles, on the distances between the centres of the nanoparticles (i.e., surface-to-surface separation between nanoparticles), and on the surface density of the polymer coating layer. Thus, the model uses the van der Waals interaction force equation, as follows [21]:

[9]
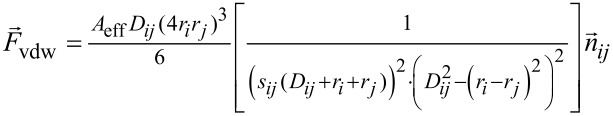


where *r_i_* and *r_j_* are spherical particle radii of the *i*-th and *j*-th nanoparticles, 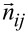 is the versor of the direction connecting the *i*-th and *j*-th particles, *D_ij_* is the distance between the centres of the *i*-th and *j*-th nanoparticles, *s_ij_* = *D_ij_* – (*r_i_* + *r_j_*) is the surface-to-surface separation between the *i*-th and *j*-th nanoparticles, and *A*_eff_ is the Hamaker effective constant for iron-oxide nanoparticles in water. When the surface-to-surface separation between two particles, *s_ij_*, is less than 1 nm, *s_ij_* is fixed at 1 nm to avoid a singularity in Equation 9.

When the normalized distances are *k*·*s_ij_* ≥ 4, the double layer electrostatic force is [21]

[10]
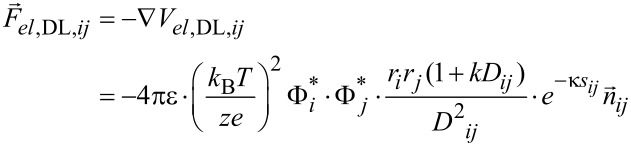


When the normalized distances are *k*·*s_ij_* < 4 [21],

[11]
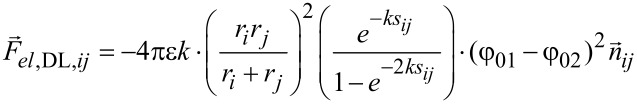


where 
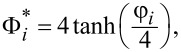
 
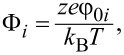
 Φ_0_*_i_* is the surface potential of the *i*-th nanoparticle at infinite separation, *z* is the ion valence, *e* = 1.6 × 10^−19^ C and *k* is the thickness of the screening ionic layer “κ”, estimated by the inverse of Debye constant.

Polymers and surfactants are usually used for steric stabilization. The model uses the following expression for the steric stabilization force [21]:

[12]
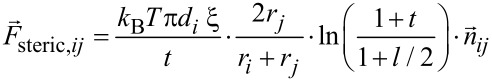


where *d_i_* = 2*r_i_*, *l* = 2*s_ij_*/*d_i_*, *t* = 2δ/*d_i_* (δ is the thickness of the surfactant layer) and ξ is the polymer surface density.

The dipolar magnetic force exerted between the magnetic moments of the *i*-th and *j*-th nanoparticles is given by [21]:

[13]
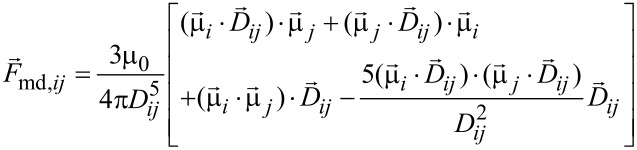


where µ_0_ is the vacuum magnetic permeability.

The random Brownian force and torque are usually modelled using the Gaussian noise [21–22]. Besides the random Brownian torque, the conservative torque acting on the nanoparticle is the magnetic torque:

[14]
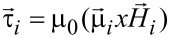


where 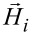 is the local magnetic field on each nanoparticle, given by Equation 4.

To solve the equations of motion numerically we use the effective Verlet-type algorithm [20–21].

### The Coffey method in an oblique magnetic field adapted to the local magnetic field on a nanoparticle

According to the literature, as a general rule, the Néel–Brown model is used to obtain the Néel relaxation time [28]. This approximation is valid only when the nanoparticles do not interact magnetostatically with one another. The external magnetic field and the dipolar magnetic field acting on the nanoparticle generate a resultant internal magnetic field 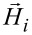 on the nanoparticle. This internal magnetic field does not generally act along the direction of the easy magnetisation axis of the nanoparticle, known as the oblique magnetic field [21–22]. This field is calculated based on Equation 4, in which the internal dipolar magnetic field is calculated by a direct sum based on Equation 3.

The nanoparticle Néel relaxation time in oblique magnetic fields is given by [21–22]

[15]
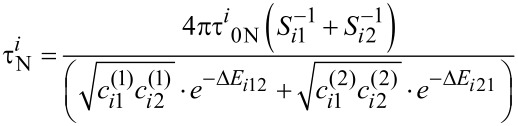


where Δ*E_i_*_12_ and Δ*E_i_*_21_ are the normalized energy barriers for the magnetic moment reorientations. The magnetisation-free diffusion time (τ*^i^*_0N_) for low damping is [21–22]

[16]
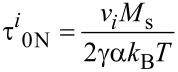


where *v_i_* is the volume of the *i*-th nanoparticle, *M*_s_ is the spontaneous magnetisation, *k*_B_ is the Boltzmann constant, *T* is the temperature, α is the damping constant, and γ is the gyromagnetic ratio.

In Equation 1,

[17]
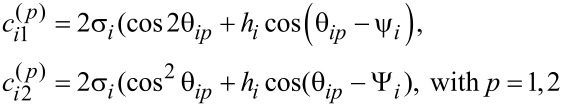


[18]
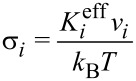


where Ψ*_i_* is the angle between 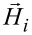 and the easy anisotropy axis of the *i*-th nanoparticle.

θ*_ip_* are the solutions of the following transcendental equation:

[19]
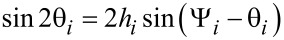


In Equation 9, θ*_i_* is the angle between the easy magnetisation and anisotropy axes of the *i*-th nanoparticle; therefore:

[20]
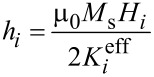


In Equation 18 and Equation 20, 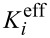 is the effective magnetic anisotropy constant of the *i*-th nanoparticle. If *h_i_* < *h_ic_*(Ψ_i_) < 1 [21–22], then

[21]
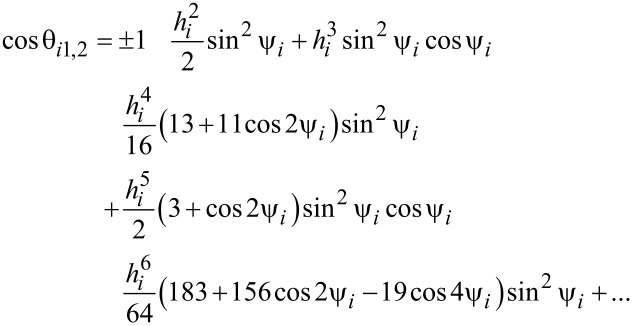


[22]
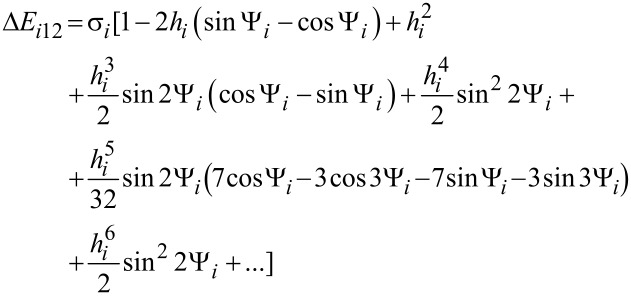


[23]
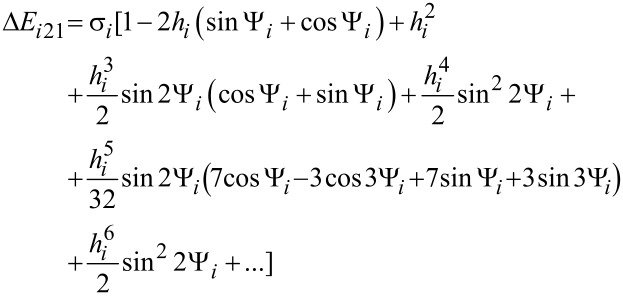


[24]
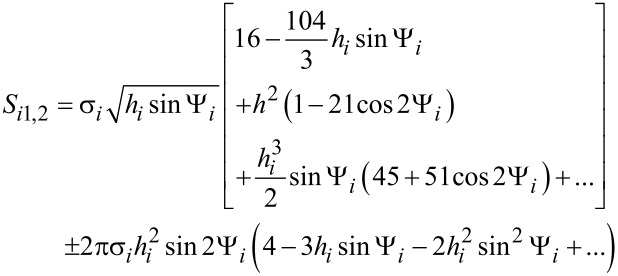


### Simulation conditions and results

For this study, we considered the case in which a colloid is electrostatically stabilised. The system is composed of water-dispersed spherical magnetite nanoparticles whose sizes follow a lognormal distribution. The Hamaker constant for magnetite in water is given as a reference value [20]. The system parameter values are given in Table 1. The external magnetic field intensity was set along the *z*-axis.

**Table 1:** The values of the parameters involved in the simulation.

Parameter name	Parameter value

number of spherical magnetite nanoparticles	50
average diameter of the nanoparticles	*d*_m_ = 10 nm
standard deviation of nanoparticle diameter	0.1·*d*_m_
volume fraction of the nanoparticles	0.05
spontaneous magnetisation	4.46 × 10^5^ A/m
dynamic viscosity	8.9 × 10^−4^ Pa·s
relative electrical permittivity	78.5
Hamaker constant for magnetite in water	39 × 10^−20^ J
temperature	298 K
thickness range of the coating layer	1–3 nm
ion concentration in solution	10^26^ ions/m^3^
ion valence	1
surface density range of the polymers (ξ)	10^16^ m^−2^–4.5 × 10^17^ m^−2^
surface charge	1.6 × 10^−15^ C
external magnetic field intensity	15 kA/m

**Figure 1:**
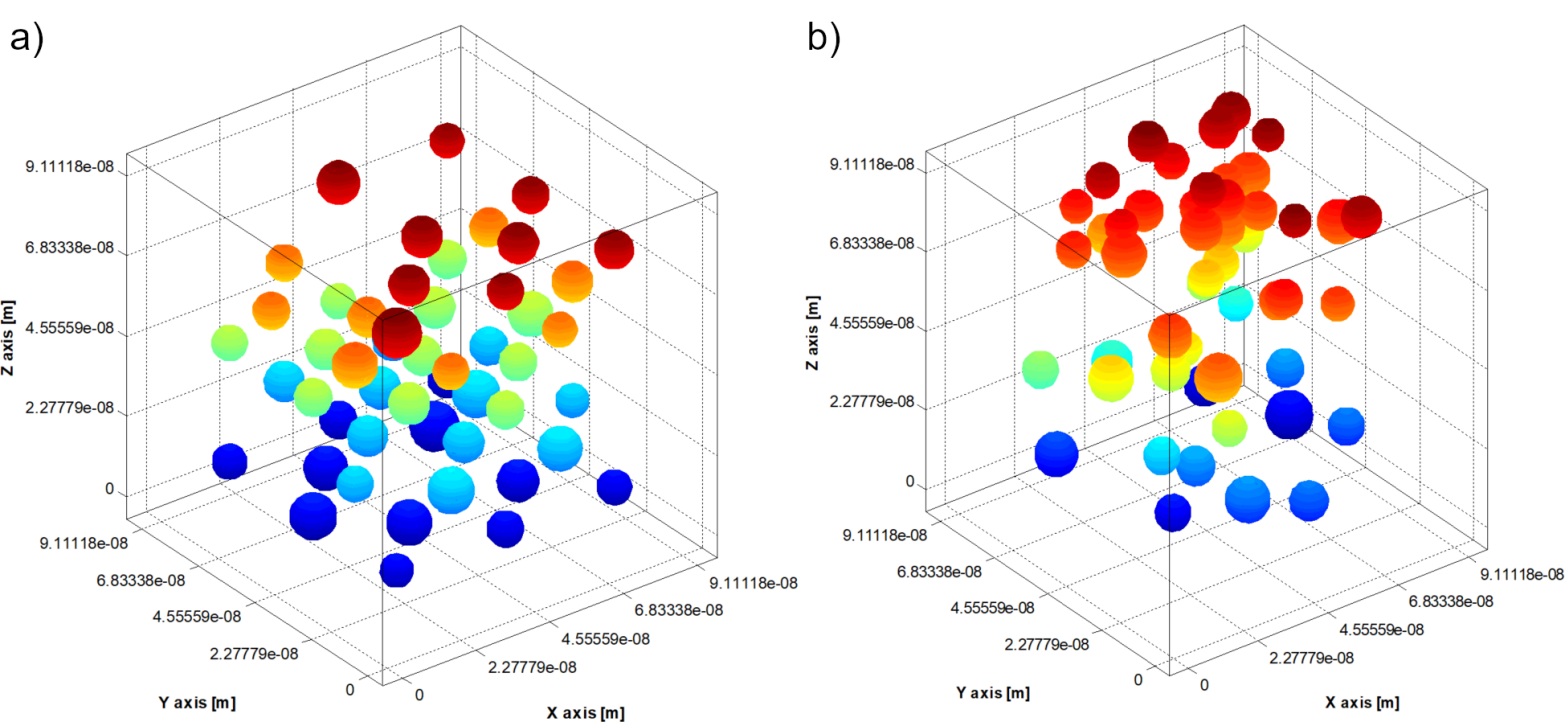
Positions of nanoparticles inside the test cube: a) initial moment; b) after 0.1 ms (thickness of the coating layer = 1 nm, polymer surface density ξ = 10^16^ m^−2^).

**Figure 2:**
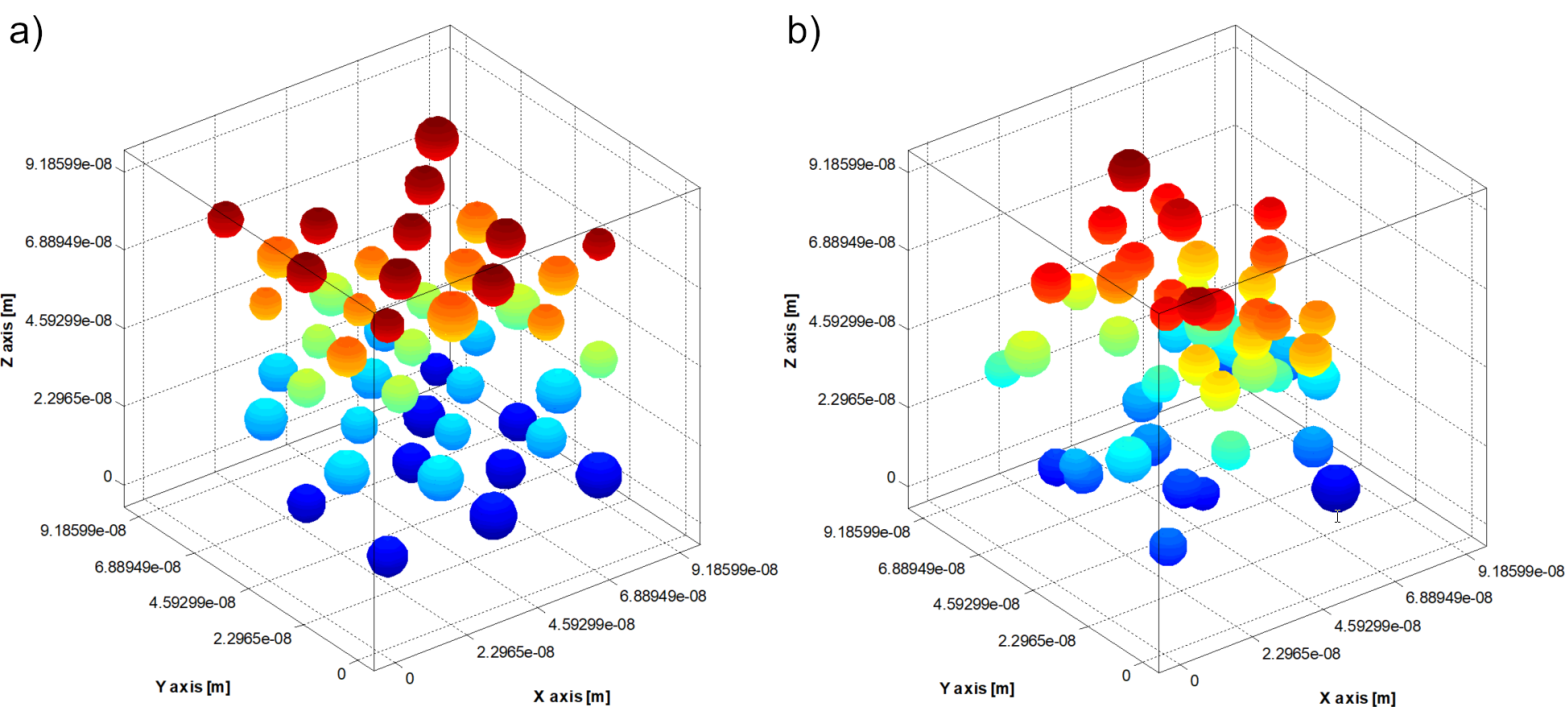
Positions of nanoparticles inside the test cube: a) initial moment; b) after 0.1 ms (thickness of the coating layer = 1 nm, polymer surface density ξ = 4.5•10^17^ m^−2^).

A random nanoparticle arrangement in a face-centred-cubic grid was initially considered. By using the Langevin dynamics stochastic method, an aggregate structure was obtained. After obtaining the aggregate structures, the effective magnetic relaxation times were calculated for the nanoparticles in the system. Then, the average effective relaxation time value was obtained as the arithmetic mean of the relaxation times. For example, Figure 1 and Figure 2 show the nanoparticle positions inside the test cube a) in the initial moment and b) after 0.1 ms (Figure 1: coating layer thickness = 1 nm, polymer surface density ξ = 10^16^ m^−2^; Figure 2: coating layer thickness = 1 nm, polymer surface density ξ = 4.5 × 10^17^ m^−2^).

**Figure 3:**
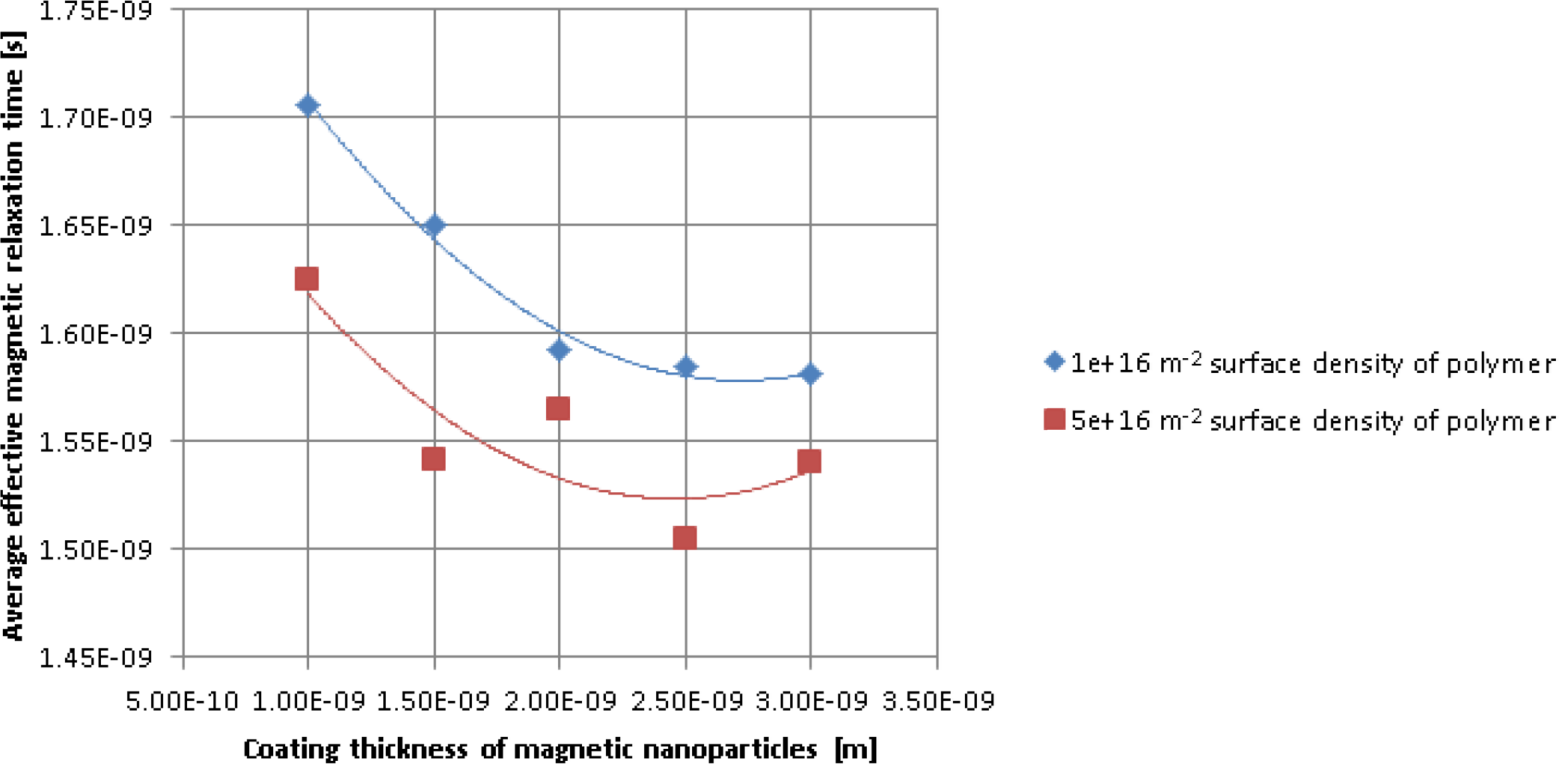
Average values of the effective magnetic relaxation time versus thickness of the nanoparticle coating layer, at low polymer surface density values.

**Figure 4:**
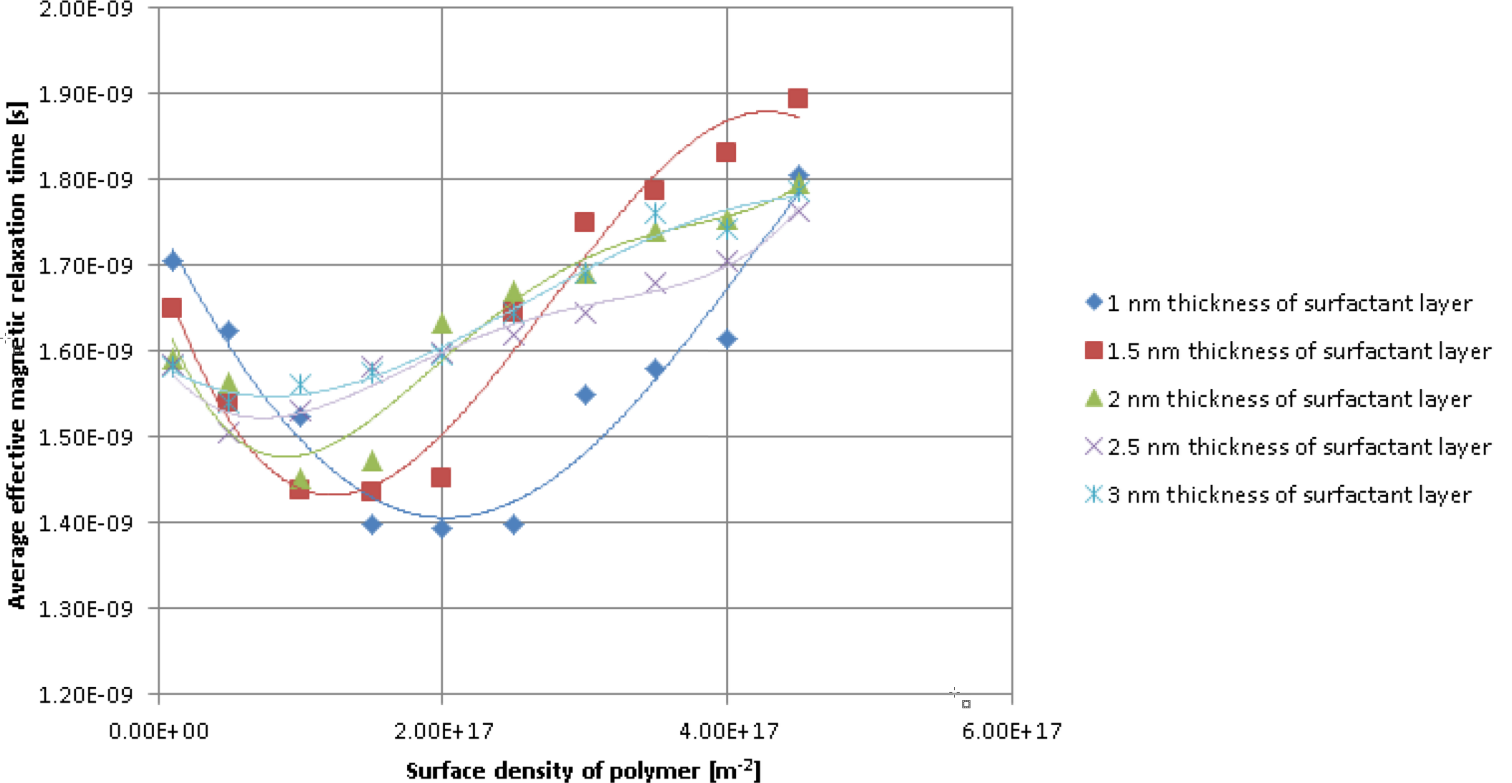
Average values of the effective magnetic relaxation time versus thickness of the nanoparticle coating layer, at high polymer surface density values.

**Figure 5:**
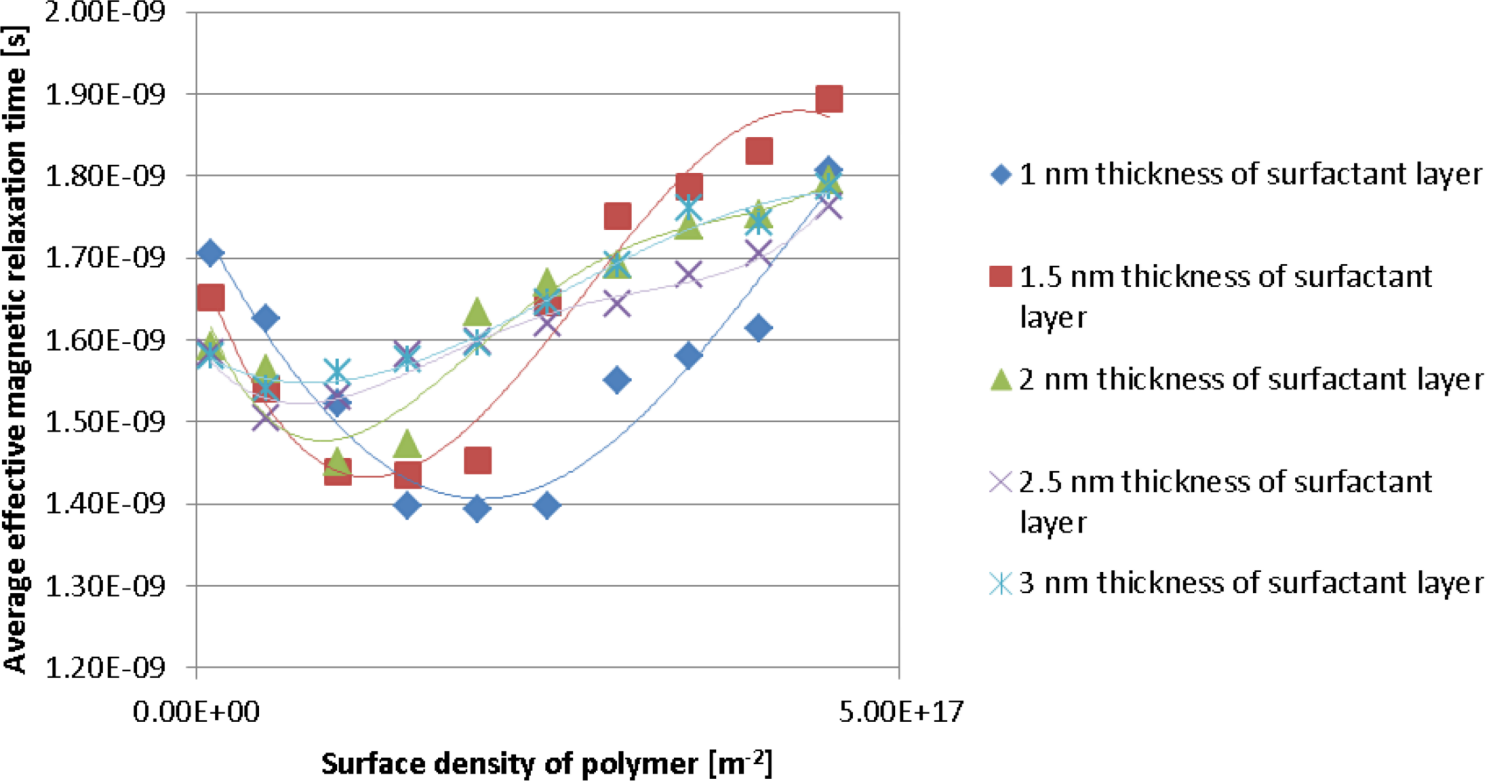
Average values of the effective magnetic relaxation time versus polymer surface density at different thickness values of the nanoparticle coating layer.

We can see in Figure 1 and Figure 2 that the polymer concentration in the nanoparticle coating influences how the nanoparticles aggregate. To study how the thickness of the nanoparticle coating layer and the polymer surface density influence the magnetic behaviour of the nanoparticles, for different values of the polymer surface density, the thickness of the nanoparticle coating layer was varied from 1 nm to 3 nm. Then, for each thickness of the coating layer, the polymer surface density was varied from 10^16^ m^−2^ to 4.5 × 10^17^ m^−2^. The results are depicted in Figures 3–5. As shown, the average effective magnetic relaxation time is affected either by the thickness of the nanoparticle coating layer or by the density of the polymer surface layer.

Figure 3 and Figure 4 show the average value of the effective magnetic relaxation time versus the thickness of the nanoparticle coating layer for low and high values of the polymer surface density, respectively. For low values of the polymer surface density in the nanoparticle coating layer (10^16^ m^−2^ and 5 × 10^16^ m^−2^), the average value of the effective magnetic relaxation time decreases with an increase in layer thickness. For high polymer surface density values, the average value of the effective magnetic relaxation time increases with the increase in coating layer thickness, then reaches a maximum value and then slightly decreases. The obtained results can be explained by the competition between the attraction forces, especially the magnetic dipolar interaction forces (Equation 13). The magnetic dipolar interaction forces are directly proportional to the magnetic moments of the particles and inversely proportional to the 5th power of the interparticle distances and the forces of repulsion, especially the steric forces (Equation 12). In addition, the magnetic dipolar interaction forces are directly proportional to the thickness of the surfactant coating layer. At low polymer surface density values, the repulsion forces, in particular the steric forces (Equation 12), are weaker. Therefore, the attraction forces predominate, especially the magnetic dipolar interaction forces which act on the nanoparticles (Equation 13). As such, the nanoparticles tend to agglomerate, resulting in a large local volumetric concentration of large nanoparticles. At high polymer surface density values, the repulsion forces, in particular the steric forces (Equation 12), are stronger in comparison to the attraction forces (e.g., the magnetic dipolar interaction forces acting on the nanoparticles, Equation 13), resulting in a smaller local nanoparticle concentration. In the extreme points (minimum, maximum), an unstable equilibrium is established between the repulsion and attraction forces. Regarding the magnetic behaviour of the superparamagnetic nanoparticle system, published studies are controversial. While some studies show that for diluted systems there is a decrease in the relaxation time when the interparticle interaction increases [30–33], others claim that the relaxation time increases when the particle concentration increases [34–35].

Figure 5 shows the effective magnetic relaxation time versus the polymer surface density for different thicknesses of the nanoparticle coating layer. Regardless of the coating layer thickness, for low polymer surface density values, the average values of both the Néel relaxation time and effective magnetic relaxation time decrease with an increase in polymer surface density until they reach a minimum value. For high values of the polymer surface density, there is an increase in the average values of both the Néel relaxation time and the effective magnetic relaxation time. The average of the minimum values of the Néel relaxation time and of the effective magnetic relaxation time increases as the thickness of the nanoparticle surfactant coating layer increases. For relatively small thickness values of the nanoparticle coating layer, the average of the minimum values of the Néel relaxation time and effective magnetic relaxation time shifts to small values of the polymer surface density, as the thickness of the surfactant coating layer of the magnetic nanoparticles increases. For larger thickness values this shift is almost unnoticeable.

This complicated dependence can also be explained by the competition between the repulsion forces (in particular the steric repulsion forces (Equation 12)) and the attraction forces (in particular the magnetic dipolar interaction forces acting on the nanoparticles (Equation 13)).

## Conclusion

Two simulation models are used in this study to investigate how the thickness of the surfactant coating layer and the density of the polymer surface layer influence both the Néel relaxation time and the effective magnetic relaxation time in a system consisting of magnetic nanoparticles suspended in a liquid matrix. To simulate the self-organization of the colloidal nanoparticles we used a stochastic method called the Langevin dynamics, which is based on an effective Verlet-type algorithm. To simulate the Néel relaxation time we used the Coffey solution in an oblique magnetic field, adapted to the local magnetic field on a nanoparticle (Equation 1). The effective magnetic relaxation time was calculated based on the Equation 11.

The numerical simulation results showed that the average values of the Néel relaxation time and the effective magnetic relaxation time are affected either by the thickness of the surfactant coating layer or by the density of the polymer surface layer.

More specifically, for small values of the polymer surface layer density, the average values of both the Néel relaxation time and the effective magnetic relaxation time decrease with an increase in coating thickness. At intermediate values of the polymer surface layer density, the average values of both the Néel relaxation time and the effective magnetic relaxation time decrease with an increase in coating thickness. Then, these relaxation time values reach a minimum, after which a slight increase occurs. At high values of the polymer surface layer density, the average values of both the Néel relaxation time and the effective magnetic relaxation time increase with an increase in coating thickness. Then, these relaxation times reach a maximum value, after which a slight decrease occurs.

It was also shown that, regardless of the coating thickness, for small values of the polymer surface layer density, the average values of both the Néel relaxation time and the effective magnetic relaxation time decrease with an increase in the polymer surface layer density. Then, these relaxation time values reach a minimum, after which, at high values of the polymer surface layer density, these relaxation time values increase again. The average of the minimum values of the Néel relaxation time and the effective magnetic relaxation time increases when the thickness of the nanoparticle surfactant coating increases. For relatively small thickness values of the nanoparticle coating layer, the average of the minimum values of the Néel relaxation time and the effective magnetic relaxation time shifts to low polymer surface density values when the surfactant coating thickness of the magnetic nanoparticles increases. For large thickness values, this shift is almost unnoticeable.

All of these behaviours related to average Néel and effective magnetic relaxation times can be explained by the competition between the repulsion and attraction forces acting on the nanoparticles.

The results presented here have the potential to be applied in several fields that use colloidal magnetic nanoparticle systems, in particular the biomedical field [36–41]. The theoretical and experimental investigation of nanoparticles with magnetic hyperthermia properties is essential for the development of alternative therapies for treating cancer in its various stages and types.
